# Tightly controlled MRTF-A activity regulates epithelial differentiation during formation of mammary acini

**DOI:** 10.1186/s13058-017-0860-3

**Published:** 2017-06-07

**Authors:** Anja Seifert, Guido Posern

**Affiliations:** Institute for Physiological Chemistry, Medical Faculty, Martin Luther University Halle-Wittenberg, 06114 Halle (Saale), Germany

**Keywords:** MRTF, MKL, EMT, Cell cycle, Morphogenesis

## Abstract

**Background:**

Myocardin-related transcription factors (MRTF) A and B link actin dynamics and mechanotransduction to gene expression. In mice, MRTF-A is involved in mammary gland differentiation, but its role in human mammary epithelial cells remains unclear.

**Methods:**

Three-dimensional cultures of human mammary epithelial MCF10A cells were used to model acinar morphogenesis. Stable MRTF-A knockdown, MRTF-A/B rescue and MRTF-A/B overexpression was established to characterize the functional role during morphogenesis using confocal microscopy and expression analysis. Breast cancer patient databases were analyzed for MRTF-A expression.

**Results:**

We showed that a precise temporal control of MRTFs is required for normal morphogenesis of MCF10A mammary acini. MRTF transcriptional activity, but not their protein amounts, is transiently induced during 3D acini formation. MRTF-A knockdown dramatically reduces acini size and prevents lumen formation. These effects are rescued by re-expression of MRTF-A, and partially by MRTF-B. Conversely, overexpression of MRTF-A and MRTF-B increases acini size, resulting in irregular spheroids without lumen and defective apico-basal polarity. These phenotypes correlate with deregulated expression of cell cycle inhibitors p21/Waf1, p27/Kip1 and altered phosphorylation of retinoblastoma protein. In MRTF overexpressing spheroids, proliferation and apoptosis are simultaneously increased at late stages, whilst neither occurs in control acini. MRTFs interfere with anoikis of the inner cells and cause an integrin switch from α6 to α5, repression of E-cadherin and induction of mesenchymal markers vimentin, Snai2 and Zeb1. Moreover, MRTF-overexpressing spheroids are insensitive to alteration in matrix stiffness. In two breast cancer cohorts, high expression of MRTF-A and known target genes was associated with decreased patient survival.

**Conclusion:**

MRTF-A is required for proliferation and formation of mammary acini from luminal epithelial cells. Conversely, elevated MRTF activity results in pre-malignant spheroid formation due to defective proliferation, polarity loss and epithelial-mesenchymal transition.

**Electronic supplementary material:**

The online version of this article (doi:10.1186/s13058-017-0860-3) contains supplementary material, which is available to authorized users.

## Background

MRTF-A (MKL1, MAL) and MRTF-B (MKL2) are members of the myocardin-related transcription factor (MRTF) family [1]. Their activity is tightly regulated by numerous mechanisms controlling rearrangement of the actin cytoskeleton [2–4]. Rho family GTPases and their effectors that control actin dynamics direct MRTF activity. MRTFs are kept in an inactive state by binding to monomeric G-actin [5]. Dissociation of this complex is crucially important to induce translocation into the nucleus and gene expression through serum response factor (SRF) binding [6]. In turn, many MRTF target genes encode components of the cytoskeleton, mechanotransduction, cell-cell and cell-matrix contacts and signaling proteins [6–8].

MRTFs have been implicated in development and tissue homeostasis including remodeling of neuronal networks, muscle cell differentiation, cardiovascular development and megakaryocytic differentiation [9]. In mice, deletion of MRTF-B provokes embryonic lethality by blocking differentiation of cardiac neural crest cells [10]. *MRTF-A* ^*−/−*^ mice have larger mammary glands, which are less organized during lactation cycles, and myoepithelial cell differentiation is defective [11, 12]. In cancer, the role of MRTFs is ambiguous. Anti-oncogenic properties of MRTF and antagonistic functions of MRTF-A on proliferative signals were reported [13, 14]. Increasing evidence, however, suggests an oncogenic function of MRTFs in controlling growth, cell motility and metastasis [7, 9, 15].

Based on these data, we investigated the functional role of MRTFs in the context of a three-dimensional (3D) organotypic culture system. We used MCF10A cells grown in matrigel, which display many properties of breast epithelial morphogenesis in vitro and serve as a valuable tool for modeling breast cancer initiation [16, 17]. Biological steps necessary for the development of MCF10A mammary epithelial acini are proliferation, epithelial polarization, signal dichotomy and apoptosis of the inner cell mass [18]. Our study shows luminal filling, polarization defects and induction of EMT markers by overexpression of MRTF-A and MRTF-B. Conversely, MRTF-A knockdown impairs acinar morphogenesis due to decreased proliferation. MRTFs are critically involved in controlling cell cycle regulators and coordinating apoptotic processes of the inner cell mass. Loss-of-function effects can be rescued by re-expression of MRTF-A, but only partially by overexpression of MRTF-B.

## Methods

### Plasmids

Details are available upon request. The SRF luciferase reporter p3D.A-Luc, the mutated p2M.A-Luc, pLVX-puro new MCS and pLVX-shRNA2-crimson have been described [19, 20]. Human full-length MRTF-A (NM_020831.4) and human MRTF-B (NM_014048.4) were amplified from Hs578T cDNA and subcloned into pLVX-puro new MCS. Annealing oligos [7] were ligated into pLVX-shRNA2-crimson to generate pLVX-shMRTFA#3 knockdown vector. Control-shRNA sequence was as follows: 5′-TTGTACTACACAAAAGTACTG-3′. The pGL4-p3D.A-Luc-Hygro vector was created by subcloning the p3D.A-Luc promoter to the pGL4.44(Luc2P/Hygro) vector (Promega, Mannheim, Germany). To generate a lentiviral pLVX-Neo-miRNA vector, the BLOCK-iT™ Pol II miRNA RNAi Expression Vector Kit (Thermo Fisher Scientific, Schwerte, Germany) was used. pcDNA™6.2-GW/EmGFP-miR vector was annealed with the following oligos to generate a new MCS (FW: 5′-TGCTTTTTGCAGGTGATGATGATGGTCGACATGATGCACCTGCTTTT-3′, rev: 5′-CCTGAAAAGCAGGTGCATCATGTCGACCATCATCATCACCTGCAAAA-3′). The *npt* gene conferring resistance to Neomycin was amplified from EGFP-C2 vector and subcloned to pcDNA™6.2-GW/EmGFP-miR vector to replace EmGFP. The resulting npt-miRNA cassette was amplified and transferred into the pLVX-puro vector to create the lentiviral pLVX-Neo-miR vector. Inserted miRNA sequences were as follows: MRTF-A-miRNA 5′-TTCCGTTTGAGATAGTCCTCT-3′ and 5′-AGGAAGAGCTGTCTGCTACTT-3′ to generate pLVX-Neo-miR-MRTF-A#1 and #2. pLVX-Neo-miR-nonsense vector was created by inserting the following oligo 5′ GAAATGTACTGCGCGTGGAGACGTTTTGGCCACTGACTGACGTCTCCACGCAGTACATTT-3′).

### MCF10A cell culture, virus production, transduction and generation of stable cell lines

MCF10A cells (American Type Culture Collection (ATCC), Manassas, VA, USA) were maintained in Dulbecco’s Modified Eagle’s Medium/Nutrient Mixture F-12 (DMEM/F12, Thermo Fisher Scientific) supplemented with 5% horse serum, 10 μg/ml insulin, 20 ng/ml epidermal growth factor (EGF), 100 ng/ml cholera toxin, 0.5 μg/ml hydrocortisone (Sigma, Taufkirchen, Germany) at 37 °C and 5% CO_2_. Stably transduced MCF10A cell populations were generated by lentiviral infection. For virus production, HEK293T cells were cotransfected by CaPO_4_ precipitation with pMD2.G (Addgene, no. 12259), psPAX2 (Addgene, no. 12260), pLVX cDNA or shRNA expression vectors. Lentivirus-containing medium was filtered, purified 48 h post-transfection by Lentivirus Concentrator (Clontech, Saint-Germain-en-Laye, France) according to the manufacturer’s instructions. Transduced MCF10A cell populations were subsequently cultured in the presence of G418 (400 μg/ml) and/or puromycin (0.5 μg/ml) (Invitrogen). Stable p3D.A expressing MCF10A were generated by transfection of pGL4-p3D.A-Luc-Hygro vector and selection with 50 μg/ml hygromycin.

### Morphogenesis assay and soft agar assay

MCF10A cells were cultured as described by Debnath et al. [16]. A single cell suspension (5000 cells per well) was plated in assay medium (growth medium with 2% horse serum, without EGF) onto eight-well chamber slides (Corning, Wiesbaden, Germany) coated with a layer of growth factor-reduced (GFR) Matrigel (Corning) and cells were overlaid with assay medium containing 5 ng/ml EGF and 2% GFR Matrigel. Morphogenesis assay were cultured for 14 days, with assay medium replaced every 2 days. Optionally at day 6 the acini were treated for 48 h with 10% collagen (Sigma). To collect acini during morphogenesis, we released acini from matrix (day 1 to 14) by 30-minute treatment with dispase (50 U/ml, BD Bioscience) and/or collagenase (50 U/ml, Sigma). The softagar assay (0.5 × 10^6^ cells) was performed as described before [21]. Phase contrast images were obtained using an EVOS FL Cell Imaging System Specs and × 10 objective (Thermo Fisher scientific).

### Immunofluorescence and microscopy

The 3D MCF10A acini and 2D cultures were fixed and stained as described by Debnath [16]. The following primary antibodies were used: anti-Ki67 (BD, Heidelberg, Germany), anti-cleaved caspase3 (Merck Millipore), anti-laminin V (abcam, Cambridge, UK), anti-MKL2 (Novus, Abington, UK), anti-MRTF-A C-19 (Santa Cruz, Heidelberg, Germany), anti-integrinα5-FITC or anti-integrinα6-PE (Miltenyi Biotech, Bergisch Gladbach, Germany). Secondary antibodies were donkey anti-mouse, anti-rabbit and anti-goat conjugated with Cy3- or Alexa 488 (Dianova, Hamburg Germany; Thermo Fisher Scientific). DNA was visualized using Hoechst 33342 (Biorad, München Germany) and F-actin was stained with Alexa Fluor 488 or Alexa Fluor 546 phalloidin (Thermo Fisher Scientific). Samples were covered with ProLong Gold antifade reagent (Thermo Fisher Scientific), and imaged using a Leica TCS SP2 AOBS confocal microscope equipped with an HPX Plan Apochromat × 63/1.4–0.6 oil objective (Leica, Wetzlar, Germany). Brightfield images of the acini central zone were taken at a Zeiss Axio Observer.Z1 equipped with a Plan-Apochromat × 20/0.8 objective and a AxioCam MrM Rev.3 CCD camera (Zeiss, Jena, Germany). Pictures were analyzed using ImageJ version 1.44p software.

### Immunoblot analysis

Immunoblot analysis was performed using 50 μg protein, according to current standard protocols. Primary antibodies were used at 1:1000 dilution (anti-Intregrin alpha5 (Merck Millipore), anti-GAPDH (Sigma), anti-MRTF-B (Novus, Abington, UK), anti-MRTF-A (homemade rabbit antiserum #79), anti-E-cadherin (Merck Millipore), anti-vimentin (Sigma), anti-Snai2, anti p27/KIP, anti-p21/Waf/Kip1 (Merck Millipore), anti-phospho-Rb S780 and anti-Rb (cell signaling, Cambridge, UK). Following overnight incubation with primary antibody, membranes were probed with IRDye 700-conjugated or IRDye 800-conjugated secondary antibodies. Proteins were detected using the Odyssey Image Scanner System (LI-COR Biosciences, Cambridge, UK). The amount of protein was measured using ImageJ version 1.44p software.

### Promoter reporter assay

Cells (5 × 10^4^) were transfected with 100 ng p3D.A-Luc or p2M.A Firefly luciferase reporter luciferase reporter construct and 10 ng pRL-TK Renilla luciferase control construct, using Lipofectamine 2000 (Thermo Fisher Scientific). Cells were serum-starved upon transfection for 24 h following treatment with 10% FCS for 7 h. Luciferase reporter assay was performed using the Dual-Glo Luciferase Assay Kit (Promega). Firefly luciferase was normalized to Renilla luciferase, or to glyceraldehyde-3-phosphate dehydrogenase (GAPDH) protein abundance for stably transfected reporter cells. To compare stably infected lines, the relative p3D.A luciferase activity was normalized to the relative p2M.A luciferase activity and is expressed as fold induction.

### RNA preparation and real-time PCR

NucleoSpin RNA XS (Macherey-Nagel, Düren, Germany) and Verso cDNA Synthesis kit (Thermo Scientific) were used according to the manufacturers’ protocols. For cDNA synthesis 1 μg of total RNA were used and cDNA was diluted to a concentration of 20 ng/μl. Real-time PCR amplification and analysis were performed using the LightCycler 480 System (Roche, Mannheim Germany) and DyNamo Flash SYBR Green qPCR kit (Thermo Scientific) according to the manufacturer’s instructions. Gene-specific primers used were: serum response factor (SRF)-FW: 5′-ACGACCTTCAGCAAGAGGAA-3′ and SRF-rev: 5′-GGAGAGTCTGGCGAGTTGAG-3′, integrin α5-FW: 5′-CTTCGGTTTACAGTCCCTCATC-3′ and integrin α5-rev: 5′-GTTGAGTCCCGTAACTCTGGTC-3′, Zeb1-FW 5′-GAAAATGAGCAAAACCATGATCCT-3′ and ZEB1-rev: 5′-CCCTGCCTCTGGTCCTCTTC-3′, GAPDH-FW: 5′-ACCCAGAAGACTGTGGATGG-3′ and GAPDH-rev: 5′-TTCTAGACGGCAGGTCAGGT-3′ and ribosomal 18S [22]. Calculations were done using the ΔΔ cycle threshold (Ct) method [23].

### Statistical analysis

Statistical analyses were usually performed using Student’s *t* test, where *p* < 0.05 was considered statistically significant, and *p* values are denoted as follows: *p < 0.05, **p < 0.01 and ***p < 0.001.

For Kaplan–Meier survival curves for patients with cancer expressing high MRTF-A, the p value scanning method was performed for 96 months of follow up using the R2 microarray and visualization platform software (http://r2.amc.nl). The Clynes dataset includes 104 mixed breast tumor samples prior to any treatment [24]. The Bertucci dataset comprises 266 tumor samples from patients with medullary breast cancer, in which the luminal A and luminal B subsets were analyzed [25]. Distribution of time to event was estimated using the Kaplan − Meier curve and the log-rank test was used to compare those distributions. Multivariate Cox models were used to estimate age-adjusted hazard ratios (HR). A two-sided *p* value <0.05 was considered to be statistically significant.

## Results

### MRTF activation during MCF10A acinar morphogenesis

**Fig. 1:**
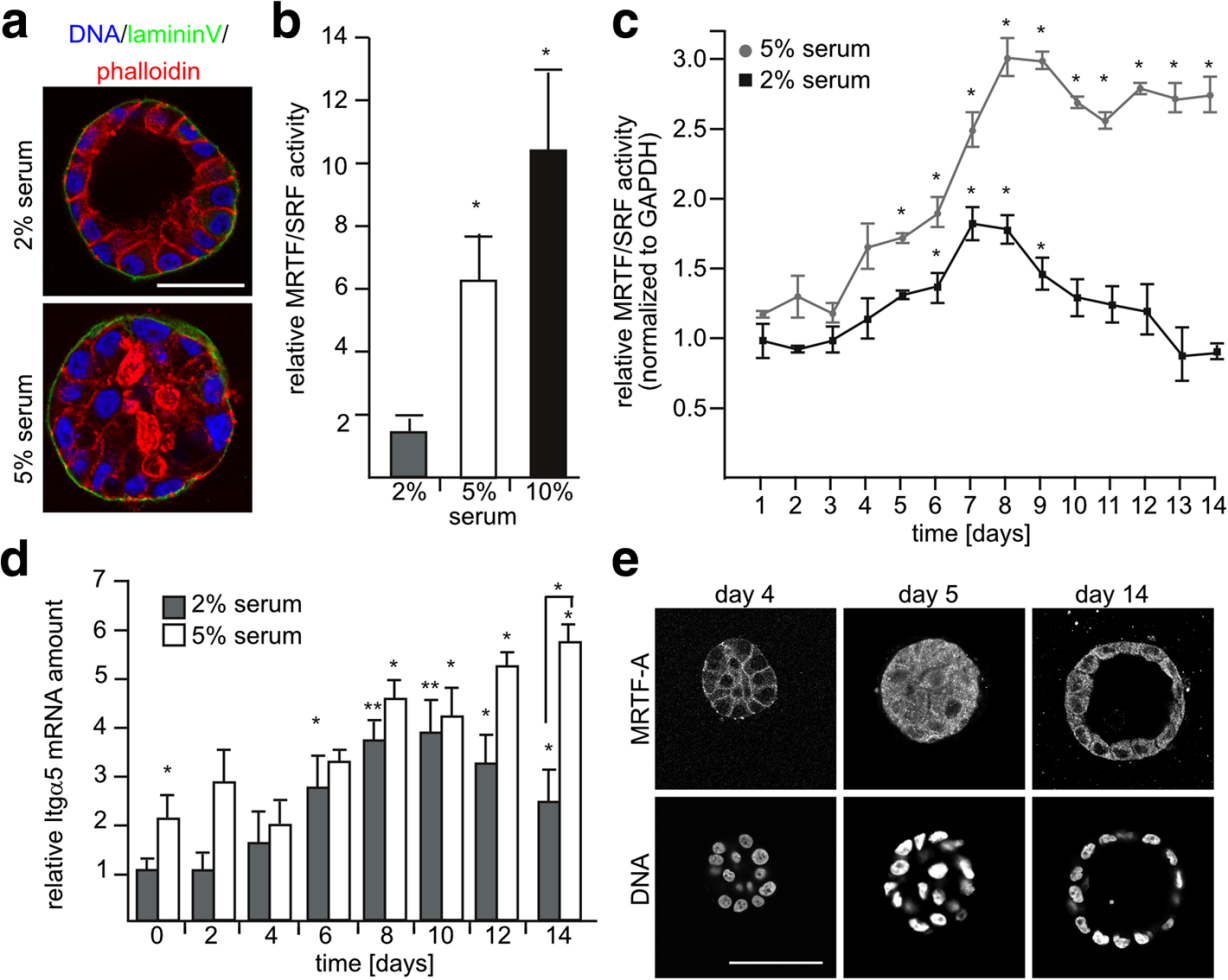
Correlation between myocardin-related transcription factor (*MRTF*)-A activity and acinar morphogenesis. MCF10A cells were grown on a solidified layer of matrigel, overlayed with medium containing 2% matrigel and the indicated amount of horse serum. **a** Phalloidin (*red*) and lamininV (*green*) staining at day 14 of acinar morphogenesis of MCF10A cells. Confocal images of representative mid plane sections. Nuclei are stained in *blue*. **b**, **c** MRTF/serum response factor (*SRF*) luciferase reporter activity. **b** MCF10A cells were transiently transfected under serum starved conditions, treated after 24 h, as indicated, and analyzed for MRTF/SRF-dependent promoter activity 7 h later. **c** Kinetics of MRTF/SRF reporter activity during 3D morphogenesis of stably transfected MCF10A cells, normalized to the amount of glyceraldehyde-3-phosphate dehydrogenase (*GAPDH*) protein. **d** Quantitative real-time PCR of integrin α5 mRNA expression during 3D-morphogenesis. *Error bars* SEM (n = 3): *p < 0.05, **p < 0.01 (Student’s *t* test). **e** MRTF-A and DNA staining at days 4, 5 and 14 of acinar morphogenesis. *Scale bar* 50 μm

To investigate processes involved in acinar morphogenesis we cultured human mammary MCF10A cells for 14 days in a 3D organotypic culture using matrigel as an extracellular matrix (ECM) [16]. We monitored acini formation using medium supplemented with different concentrations of horse serum. Lumen formation and epithelial polarization with prominent lamininV localization at the basal side of the acini was readily observed at 2% serum concentration (Fig. 1a). In contrast, luminal clearance was impaired after cultivation in medium containing an elevated serum level. Subsequently, we measured a promoter luciferase reporter construct, which depends on the activity of the MRTF/SRF transcription factor module, a major mediator of the serum response pathway. In 2D MCF10A cultures, the MRTF/SRF activity correlated with increasing serum concentration after 7 h of stimulation (Fig. 1b).

To determine whether MRTF-SRF transcriptional activity is also affected during acinar morphogenesis, we extracted acini from 3D cultures and measured either the induction of a stably transfected MRTF/SRF luciferase reporter or the expression of the MRTF-A target gene integrin α5. The temporal analysis showed serum-dependent differences in MRTF/SRF activity and target gene induction kinetics during acini formation (Fig. 1c, d). In acini-permissive 2% serum conditions, MRTF/SRF reporter activity was significantly induced between days 6 and 9, and decreased thereafter (Fig. 1c). In 5% serum-containing culture conditions, however, a sustained increase in MRTF/SRF activity was observed. In line with the reporter, integrin α5 mRNA expression was only transiently induced threefold to fourfold between days 6 and day 10 of acinar morphogenesis in 2% serum (Fig. 1d). At 5% serum, twofold to threefold higher basal expression of integrin α5 was measured, followed by a small but sustained increase until day 14 (Fig. 1d).

However, the changes in transcriptional activity during the time of acinar morphogenesis were not reflected by differences in the amoun of MRTF-A and MRTF-B protein, which remained at constantly low levels compared to fibroblasts (Additional file 1: Figure S1). Thus, we investigated the nuclear accumulation of MRTF-A, which is often indicative of enhanced MRTF/SRF signaling. Despite difficulties in staining 3D acini, nuclear accumulation of MRTF-A was observed initially on day 5 of acinar morphogenesis and declined toward day 14 (Fig. 1e). These results suggest that a precise temporal balance of MRTF-dependent gene expression is critical for acinar morphogenesis.

### Overexpression of MRTF-A and MRTF-B causes luminal filling, polarization defects and enlarged acini formation

**Fig. 2:**
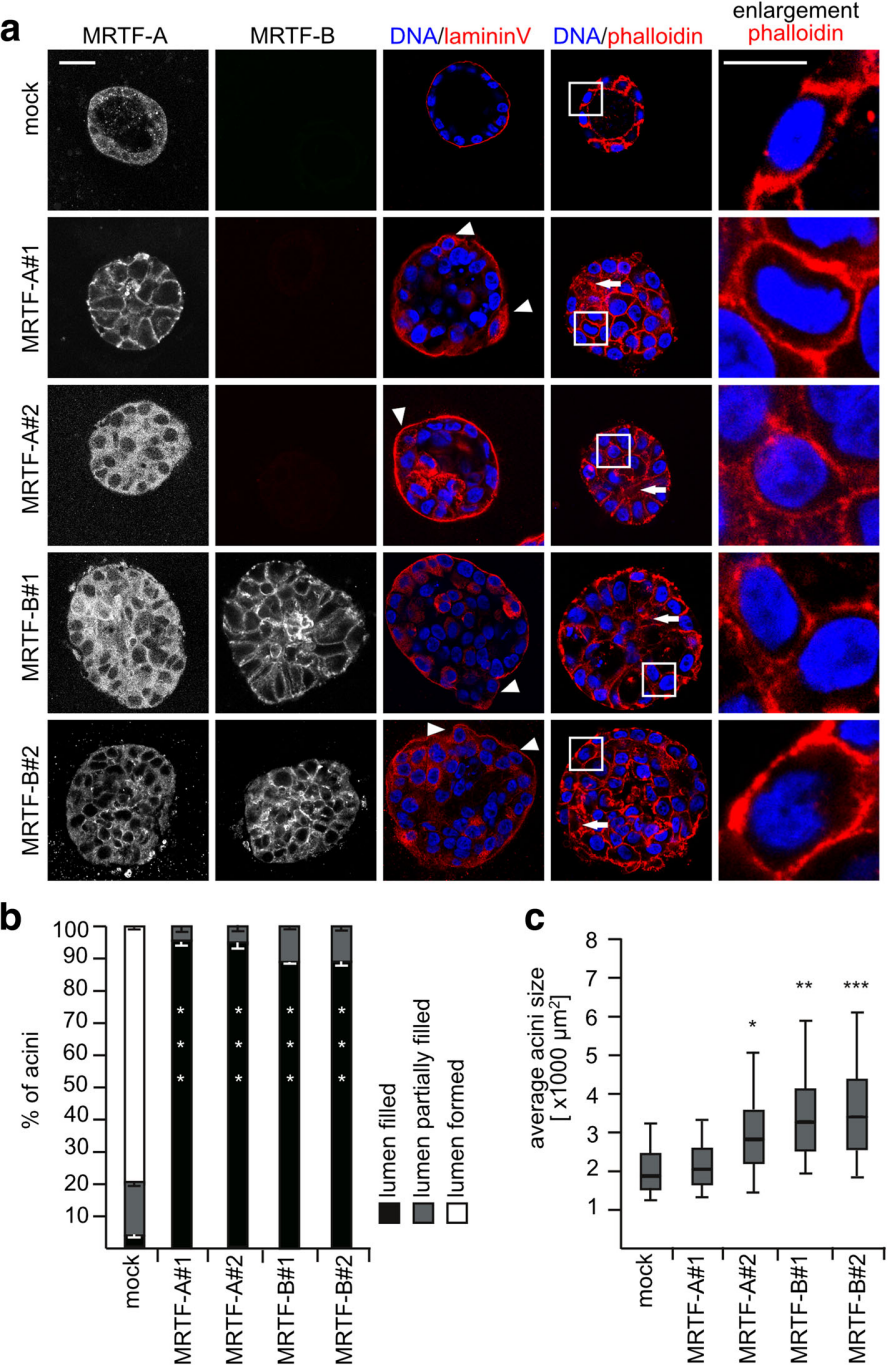
Luminal filling and enhanced acini size observed upon myocardin-related transcription factor (*MRTF*) overexpression. Cells were grown on matrigel in medium containing 2% matrigel and 2% horse serum for 14 days as before. **a** Immunofluorenscence staining of acini formed by the indicated cell lines. Representative mid plane sections are shown for MRTF-A, MRTF-B, lamininV (*red*), phalloidin (*red*) and DNA (*blue*). *Scale bar* 20 μm. **b** Quantification of lumen formation detected from confocal images of 30 acini from three independent experiments. **c** Acini size measured from bright field images. Shown is the average mid plane area per cell line for at least 100 acini in three independent experiments. The *box* represents the interquartile range and the *middle line* represents the median; the *whiskers* extend to the 5th and 95th percentiles: *p < 0.05, **p < 0.01, ***p < 0.001 (Student’s *t* test)

To specify the effects of MRTF activity on acinar morphogenesis, MCF10A cell lines stably overexpressing MRTF-A or MRTF-B were created by lentiviral transduction. Two independent pools of stable MCF10A cell lines were generated, displaying an obvious MRTF-B expression or a twofold increase in MRTF-A (Additional file 2: Figure S2A). These cell lines showed elevated basal MRTF/SRF reporter activity and significantly greater induction by addition of 10% horse serum (Additional file 2: Figure S2B). Mock-transduced MCF10A control cells in acinar morphogenesis assays showed a properly formed acinar structure at day 14, with a hollow lumen surrounded by a monolayer of epithelial cells (Fig. 2a, top panel). As expected for apico-basal polarization, the nuclei were flattened against the lamininV-positive basement membrane, which smoothly surrounded the entire acini. Cortical F-actin was localized predominantly at the lateral contact zones and at the apical side of the control cells.

In contrast, overexpression of MRTF-A or MRTF-B caused a prominent luminal filling phenotype and an increased size after 14 days of culture (Fig. 2a, lower panels). False localization of lamininV throughout the spheroids was observable, illustrating defects in polarization. Cells bulged outwards (Fig. 2a marked by arrowhead) and destroyed the evenly rounded structure seen in intact acini. Compared to control cells the cytoskeleton in some parts appeared to be disturbed (Fig. 2a, marked by arrows). In MRTF-B-overexpressing spheroids, cortical actin was also localized to the basal side. The quantification revealed that the luminal filling was observed in more than 85% of the spheroids overexpressing MRTF-A or MRTF-B, compared to less than 5% in control acini (Fig. 2b). Additionally, MRTF-A#2, MRTF-B#1 and MRTF-B#2 cell lines formed significantly larger (mean 1.5-fold to 2.0-fold) spheroids than mock-transduced control cells (Fig. 2c). Together, these results indicate that activation of MRTF-A or MRTF-B impairs acini formation and epithelial polarization.

### Knockdown of MRTF-A disrupts acini formation

Further, we investigated the function of MRTF-A during acinar morphogenesis by a loss-of-function approach. MRTF-A knockdown was achieved by three different shRNA (shMRTF-A#1-3) expression constructs using two different vector systems compared to cells expressing control shRNA (shCtrl). Additionally, we generated cell lines re-expressing MRTF-A subsequent to the knockdown (sh#1 + MRTF-A, sh#2 + MRTF-A). In addition, we produced cell lines overexpressing MRTF-B in MRTF-A knockdown cells (sh#1 + MRTF-B, sh#2 + MRTF-B) to examine functional redundancy of MRTF-A and MRTF-B.

**Fig. 3:**
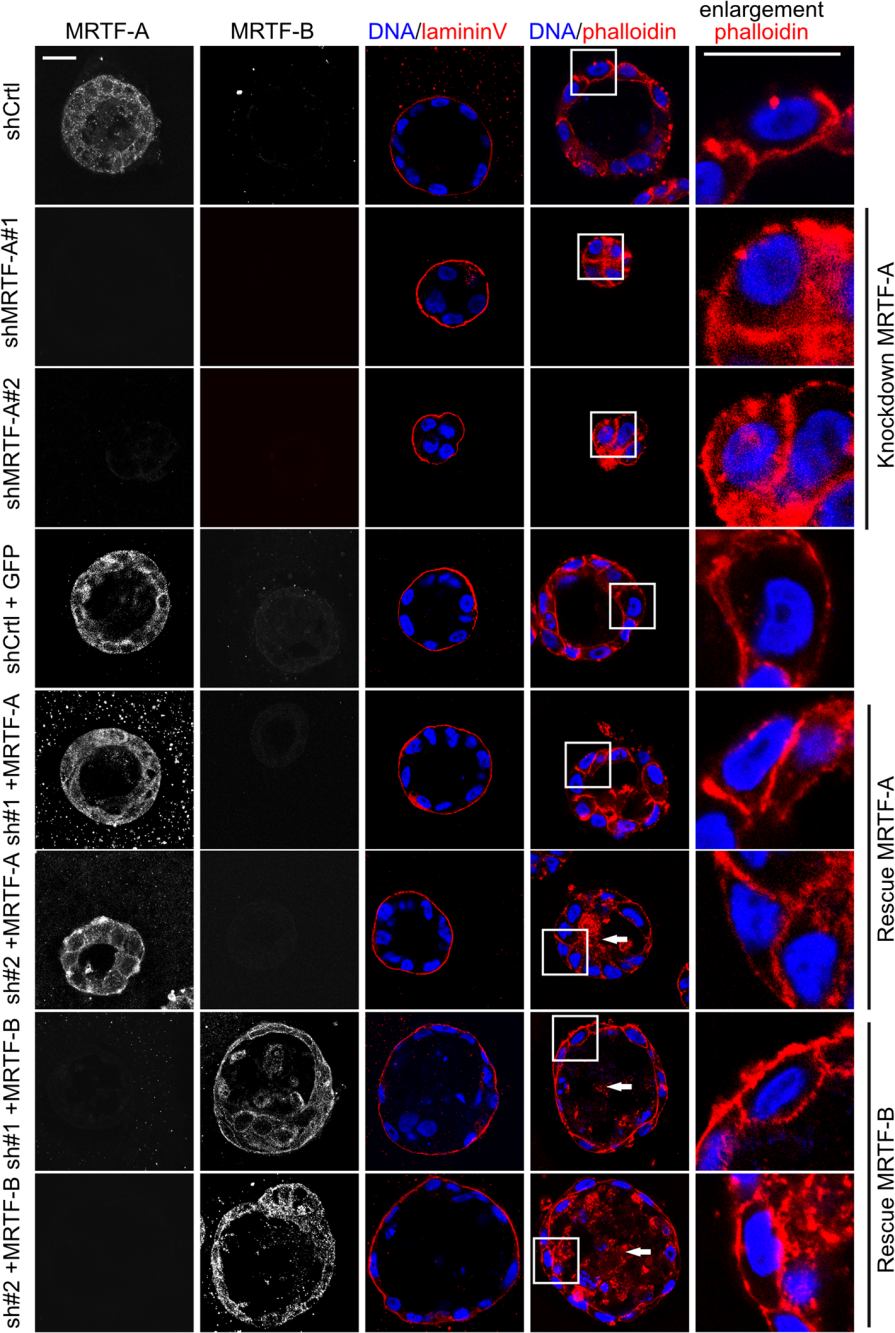
MCF10A acini formation is myocardin-related transcription factor (*MRTF*) dependent. Stably transduced cells harboring control or MRTF-A knockdown (*shCtrl*, *shMRTF-A#1*, *shMRTF-A#2*), recue of MRTF-A (*sh#1 + MRTF-A*, *sh#2 + MRTF-A*), rescue with MRTF-B (*sh#1 + MRTF-B*, *sh#2 + MRTFB*) or control rescue (*shCtrl + GFP*) were cultured in matrigel for 14 days as before. Immunofluorenscence staining of MRTF-A, MRTF-B, lamininV (*red*), phalloidin (*red*) and DNA (*blue*) from representative confocal mid plane sections are shown. *Scale bar* 20 μm

MRTF-A knockdown cell lines formed grossly disorganized acini of reduced size without any lumen at day 14 (Fig. 3). The resulting structures consisted of very few cells, and consequently, all cells had direct contact to the ECM. No changes were observed in the localization of the polarization marker lamininV (Fig. 3, Additional file 3: Figure S3A). Re-expression of MRTF-A in shMRTF-A cells largely rescued the normal acinar morphogenesis, although some deposits of apoptotic cells were observed on phalloidin staining. These experiments demonstrate that acini formation, in particular, proliferation in 3D matrigel, requires MRTF-A.

Interestingly, expression of MRTF-B in MRTF-A knockdown cells was not able to fully restore proper acinar morphogenesis (Fig. 3, lower panels). LamininV was located at the basal side, but the acini were larger. Moreover, in the cells that contact the ECM, the cortical F-actin was not restricted to the lateral sides. In the lumen disturbed phalloidin-positive structures of dead and living cells were found observed (Fig. 3).

**Fig. 4:**
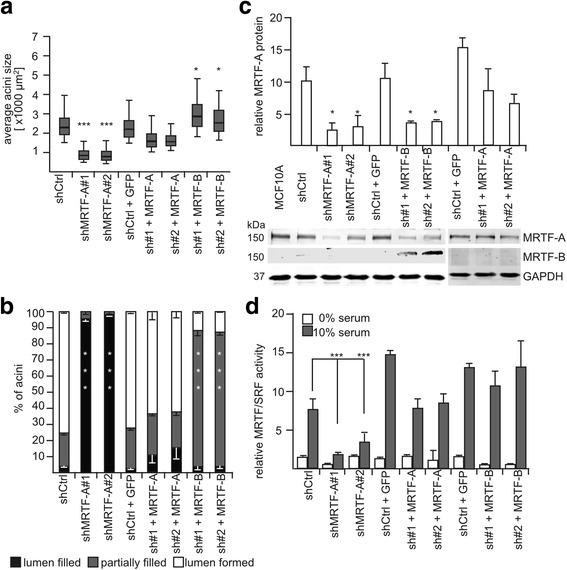
Quantification of acini size, luminal filling and is myocardin-related transcription factor (*MRTF*) activity. **a** Quantification of acini size on bright field images. The size of at least 100 acini was measured, each from three independent experiments as described. The *box* represents the interquartile range and the *middle line* represents the median; the *whiskers* extend to the 5th and 95th percentiles. **b** Quantification of lumen formation detected from confocal images of 30 acini each from three different experiments. **c** Relative protein expression of MRTF-A and MRTF-B in the knockdown and recue cell lines indicated. Shown is the mean of three independent experiments. A representative immunoblot of MRTF-A and MRTF-B from 2D cell cultures is shown below. **d** Indicated cell lines were transiently transfected under serum-starved conditions, treated after 24 h, as indicated, and analyzed for MRTF/serum response factor activity. *Error bars* SEM (n = 3): *p < 0.05, ***p < 0.001 (Student’s *t* test). *shCrtl* shRNA control, *GAPDH* glyceraldehyde-3-phosphate dehydrogenase, *GFP* green fluorescent protein

The quantification illustrated the changes in acini size observed by immunofluorescence microscopy (Fig. 4a). The size of MRTF-A knockdown structures was reduced twofold to threefold, whilst re-expression of MRTF-A in shMRTF-A cells essentially restored acini size. In contrast, expression of MRTF-B in MRTF-A knockdown cells increased the acini to a size larger than that of the controls. The quantification of lumen formation revealed that more than 90% of MRTF-A knockdown acini contain no lumen, likely caused by the decreased acini size (Fig. 4b, Additional file 3: Figure S3B). Re-expression of MRTF-A in MRTF-A knockdown cells rescued this effect, whilst expression of MRTF-B in MRTF-A knockdown acini possessed a lumen partially filled with living cells and cell remnants in approximately 80% of the acini (Fig. 4b). This suggests a partially non-redundant function of MRTF-A and MRTF-B during acinar morphogenesis.

Molecular characterization revealed that MRTF-A expression was reduced to 20% in knockdown cells compared to control cells (Fig. 4c). This was accompanied by reduced inducibility of the MRTF/SRF reporter to a level comparable to the serum-starved activity in control cells (Fig. 4d, Additional file 3: Figure S3). In MRTF-A rescue cells, the MRTF-A expression and reporter activity were essentially restored to the level of shCtrl cells. In MRTF-B overexpressing MRTF-A knockdown cells, a fully restored MRTF/SRF-dependent promoter activity upon serum stimulation was observed.

### MRTFs affect cell cycle regulators

To investigate the reasons for the observed changes in MRTF knockdown acini, we analyzed proliferation and cell cycle regulators. We stained 3D cultures with the proliferation marker Ki67 at day 4 to visualize proliferating cells at early stages of acinar morphogenesis. Additionally, we analyzed the cyclin-dependent kinase inhibitors (CKI) p21/Waf1, p27/Kip1 and the amounts of total and phosphorylated retinoblastoma protein (Rb) by immunoblotting of acini recovered at day 14.

**Fig. 5:**
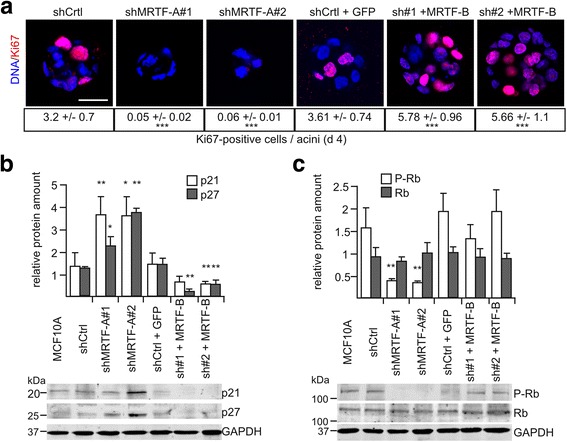
Knockdown of myocardin-related transcription factor (*MRTF*)-A impairs proliferation and cell cycle. **a** Knockdown and recue cell lines were cultured in matrigel for 4 days and stained for the proliferation marker (*scale bar* 20 μm). Quantification of Ki67-positive cells per acini, determined from 30 acini in three different experiments. **b**, **c** Proteins from acini recovered at day 14 were immunoblotted for p21 and p27 (**b**) or phospho-retinoblastoma protein (*P-Rb*) and retinoblastoma protein (*Rb*) (**c**). *Upper panels* show normalized protein content, and a representative immunoblot is shown below. *Error bars* SEM (n = 3): *p < 0.05, **p < 0.01 (Student’s *t* test). *SRF* serum response factor. *shCrtl* shRNA control, *GAPDH* glyceraldehyde-3-phosphate dehydrogenase, *GFP* green fluorescent protein

At day 4, we hardly detected any Ki67-positive cells in any of the three MRTF-A knockdown acini in contrast to control acini (Fig. 5a, Additional file 3: Figure S3E). Instead, increased expression of p21/Waf and p27/Kip was visible, whilst Rb was hypophosphorylated (Fig. 5b, c). In MRTF-A rescue acini Ki67 was expressed to a similar level when compared to the control (Additional file 4: Figure S4). This suggests that knockdown of MRTF-A impairs proliferation by upregulating CKIs and preventing Rb phosphorylation (S780). Interestingly, expression of MRTF-B in the MRTF-A knockdown cells led to an increase of Ki67-positive cells beyond the level of parental or mock-transduced control acini (Fig. 5a). Similarly, p21/Waf and p27/Kip expression were reduced to almost undetectable levels (Fig. 5b, c). This suggests that the increased size of MRTF-A knockdown acini is partially rescued by MRTF-B and may result from elevated cell proliferation based on reduced CKI expression.

**Fig. 6:**
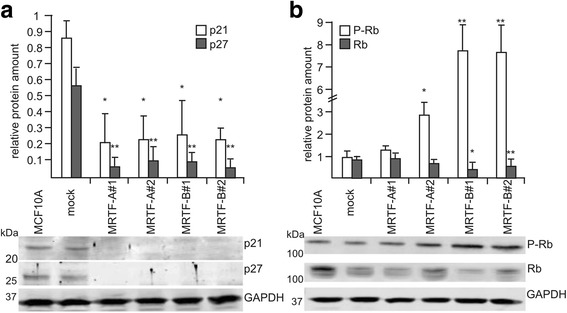
Myocardin-related transcription factor (*MRTF*) overexpression affects cell cycle regulators. **a**, **b** p21, p27, phospho-retinoblastoma protein (*P-Rb*) and retinoblastoma protein (*Rb*) expression at day 14 of acinar morphogenesis of the indicated MRTF-overexpressing cell lines. Shown are normalized protein content and a representative immunoblot below. *Error bars* SEM (n = 3): *p < 0.05, **p < 0.01 (Student’s *t* test). *GAPDH* glyceraldehyde-3-phosphate dehydrogenase

Thus, we revisited the MRTF-A or MRTF-B overexpressing cells to analyze proliferation, apoptosis and cell cycle regulators in their lumen-filled acini. Strikingly, MRTF-A and MRTF-B overexpression caused approximately 90% reduction of p27/Kip protein and 75% reduction of p21/Waf protein during acinar morphogenesis (Fig. 6a). Except for the MRTF-A#1 line, all spheroids showed significantly higher Rb phosphorylation (S780), although total Rb was reduced in the MRTF-B-overexpressing spheroids (Fig. 6b). These results suggest that the enhanced size of the filled MRTF overexpressing spheroids is due to dysregulation of cell cycle checkpoints.

**Fig. 7:**
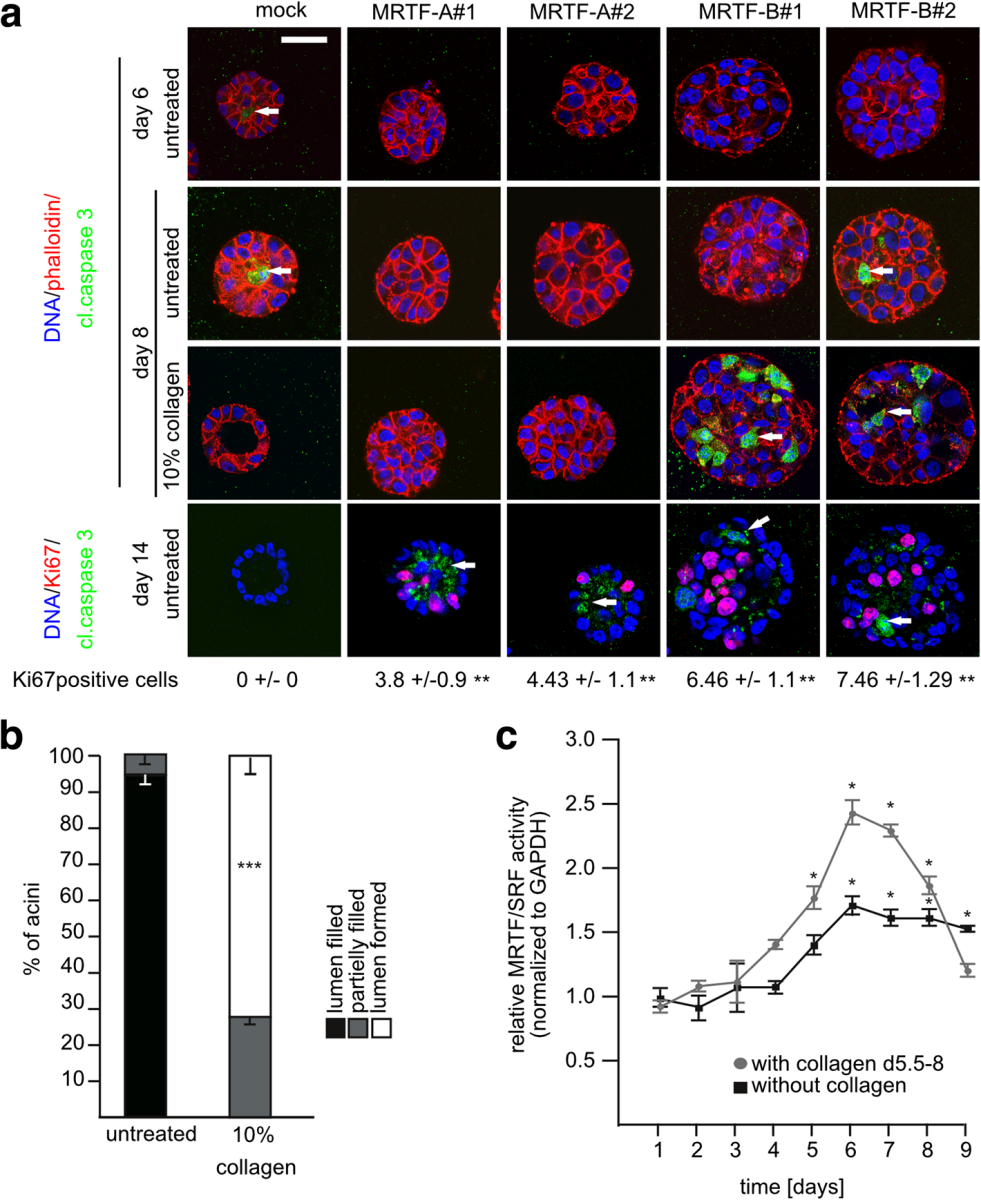
Implication of myocardin-related transcription factor (*MRTF*) during luminal apoptosis in normal and accelerated formation of acini. **a** Cells were grown in matrigel with 10% collagen for the last 2 days if indicated. Phalloidin (*red*), cleaved caspase3 (*green*), Ki67 (*red*) and DNA (*blue*) staining of representative mid plane sections is shown. Quantification of Ki67-positive cells per acini; 30 acini each were analyzed. *Scale bar* 20 μm. **b** Quantification of lumen formation of mock-transfected control acini at day 8. Analysis of 30 acini each, with or without prior collagen treatment. **c** Time course of MRTF/serum response factor luciferase reporter (*SRF*) activity during 3D morphogenesis of stably transfected MCF10A reporter cells, normalized to glyceraldehyde-3-phosphate dehydrogenase (*GAPDH*) protein abundance. *Error bars* SEM (n = 3): *p < 0.05, **p < 0.01, ***p < 0.001 (Student’s *t* test)

Further, we investigated apoptosis of the inner cells at day 6 of acini formation using cleaved caspase 3 as an apoptosis marker. Whereas control cells clearly showed cleaved caspase 3 staining of the inner cells, no cleaved caspase 3 was detectable in spheroids overexpressing either MRTF-A or MRTF-B (Fig. 7a). To determine if we can enforce lumen formation and apoptosis in MRTF-overexpressing spheroids, we experimentally stiffened the matrix by collagen addition. Whilst permanently stiffened gels disrupted acini formation (data not shown), transient addition of 10% collagen at day 5.5 for 2 additional days indeed accelerated lumen formation in control acini, with lamininV properly located to the basal side (Fig. 7a, and data not shown). Interestingly, in collagen-treated MRTF-A-overexpressing spheroids there was still no lumen formed, whilst 70% of the collagen-treated control acini exhibited complete lumen formation under these conditions (Fig. 7a, b). This indicates that overexpressed MRTF-A efficiently prevents apoptosis or anoikis of cells that are not in contact with the basal lamina.

Similarly, MRTF-B-overexpressing spheroids lacked lumen formation in the enforced conditions, although some cleaved caspase-3-positive cells were observed. However, the localization of cleaved caspase 3 was not restricted to the inner cells, as seen in the controls (Fig. 7a). At day 14 of normal morphogenesis, more than 80% of MRTF-A or MRTF-B overexpressing spheroids possessed cleaved caspase 3 and ascertainable Ki67-positive cells distributed throughout the spheroids (Fig. 7a, Additional file 5: Figure S5). Taken together, this shows that both aberrant proliferation and apoptosis occurred concomitantly in MRTF-overexpressing spheroids, finally leading to filled lumen with deposits of apoptotic cells.

The involvement of MRTF activity in accelerated luminal clearance by collagen treatment was analyzed during morphogenesis of stably transfected MRTF/SRF reporter cells. Two days collagen treatment markedly intensified the induction of MRTF/SRF-luciferase reporter activity, compared to normal acini culture conditions at days 6 to 7 (Fig. 7c). Until day 9, the collagen-enhanced MRTF/SRF activity reverted to the basal level found at days 1 to 3. As before, these results are consistent with an important role of MRTF-induction and/or shutdown during acini formation, and indicate that endogenous MRTFs transduce mechanical signals from the ECM.

### Persistent MRTF overexpression/activity causes EMT in MCF10A cells

**Fig. 8:**
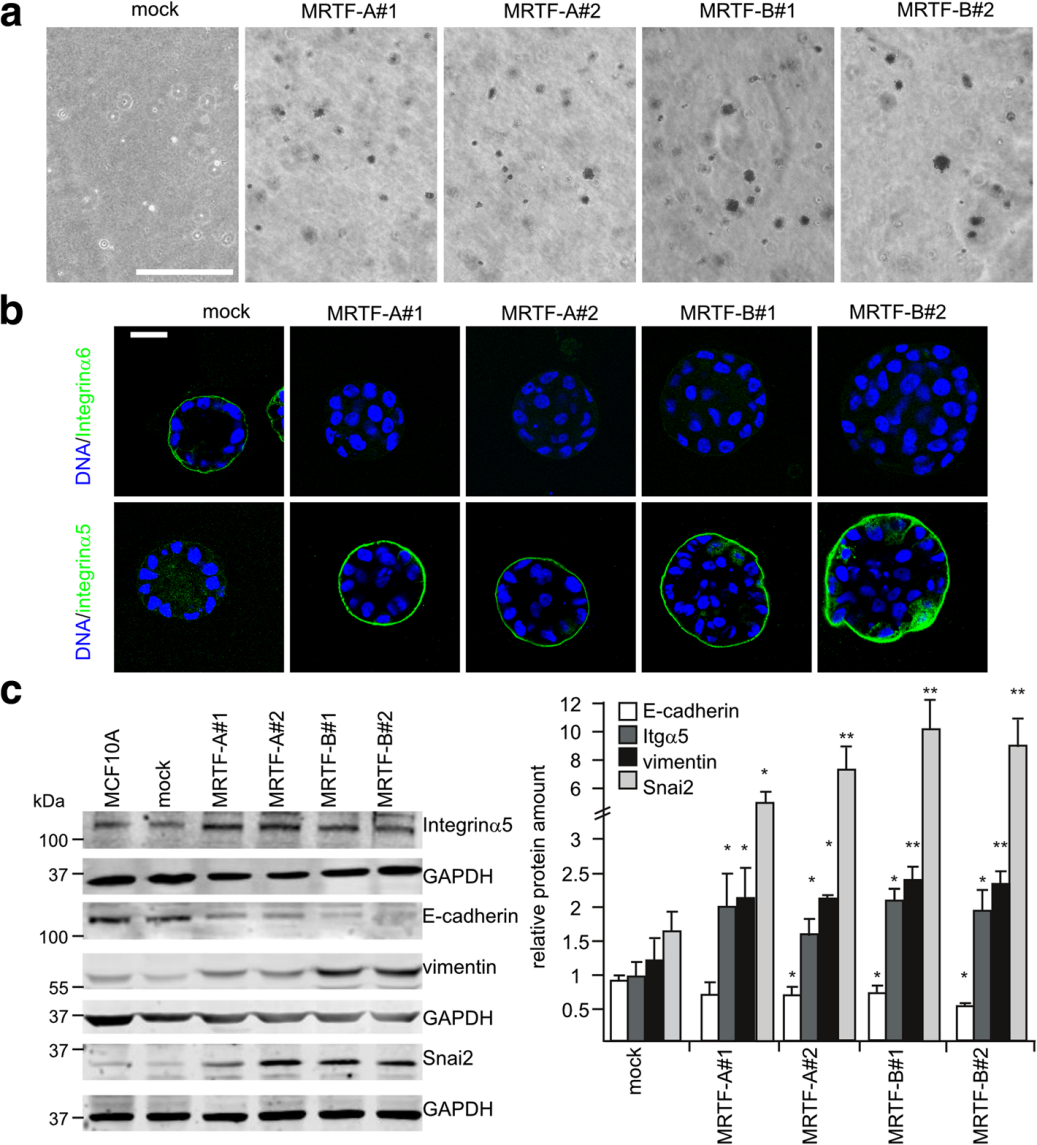
Anchorage-independent growth and epithelial-mesenchymal transition marker expression regulated by myocardin-related transcription factor (*MRTF*). **a** MRTF-overexpressing cells were grown embedded in agarose-matrix. Bright field micrographs were taken after 6 weeks. *Scale bar* 400 μm. **b** Integrin α5 and α6 staining of 14-day-old acini formed by the indicated cell lines. *Scale bar* 20 μm. **c** Protein expression of integrin α5, E-cadherin, vimentin and Snai2/Slug in 2D cultures of MRTF-overexpressing cell lines. Representative immunoblot (*left*) and quantification of normalized amount of protein (*right*) is shown. *Error bars* SEM (n = 3): *p < 0.05, **p < 0.01 (Student’s *t* test). *GAPDH* glyceraldehyde-3-phosphate dehydrogenase

The immunofluorescence studies suggest that cell survival gains independence of ECM contact in MRTF overexpression in the spheroids. Consequently, we examined anchorage-independent growth. MCF10A cells were unable to grow independent of anchorage and formed no colonies in soft agar, in line with previous reports [26]. In contrast, MRTF-overexpressing cells survived and formed small colonies upon 6 weeks of cultivation in soft agar, demonstrating their gained capacity for anchorage-independent growth (Fig. 8a). We determined expression and localization of integrin α5 and integrin α6 to investigate if an integrin switch caused the observed growth behavior. The fibronectin receptor integrin α5 is a known MRTF-A target gene, whilst integrin α6 is an epithelial marker involved in laminin binding. Strikingly, the localization of integrin α6 to the acini outer side was lost in both spheroids overexpressing MRTF-A or MRTF-B (Fig. 8b). In contrast, integrin α5 was predominantly located at the basal side of the MRTF-overexpressing spheroids, whereas control acini showed only diffuse and weak integrin α5 staining. Moreover, immunoblotting revealed that integrin α5 expression was induced twofold in MRTF gain-of-function acini (Fig. 8c).

The observed integrin switch and the anchorage independence were indicative of altered cell adhesion and/or a transition from an epithelial to an invasive, mesenchymal cell fate. Thus, we investigated the expression of additional epithelial and mesenchymal markers in acini/spheroids overexpressing MRTF-A or MRTF-B. We detected a remarkable reduction in the epithelial adherens junction protein E-cadherin (Fig. 8c). Concomitantly, the mesenchymal marker proteins vimentin and Snai2 were induced approximately twofold and sixfold to tenfold in MRTF overexpressing cells, respectively. Whereas Twist mRNA remained unchanged, Zeb1 mRNA expression was increased twofold to threefold (Additional file 6: Figure S6). Collectively, these results implicate MRTF function in dedifferentiating the mammary epithelial architecture, and suggest an important regulatory role of MRTFs during acini formation.

### Increased expression of MRTF-A and target genes correlates with reduced breast cancer survival

**Fig. 9:**
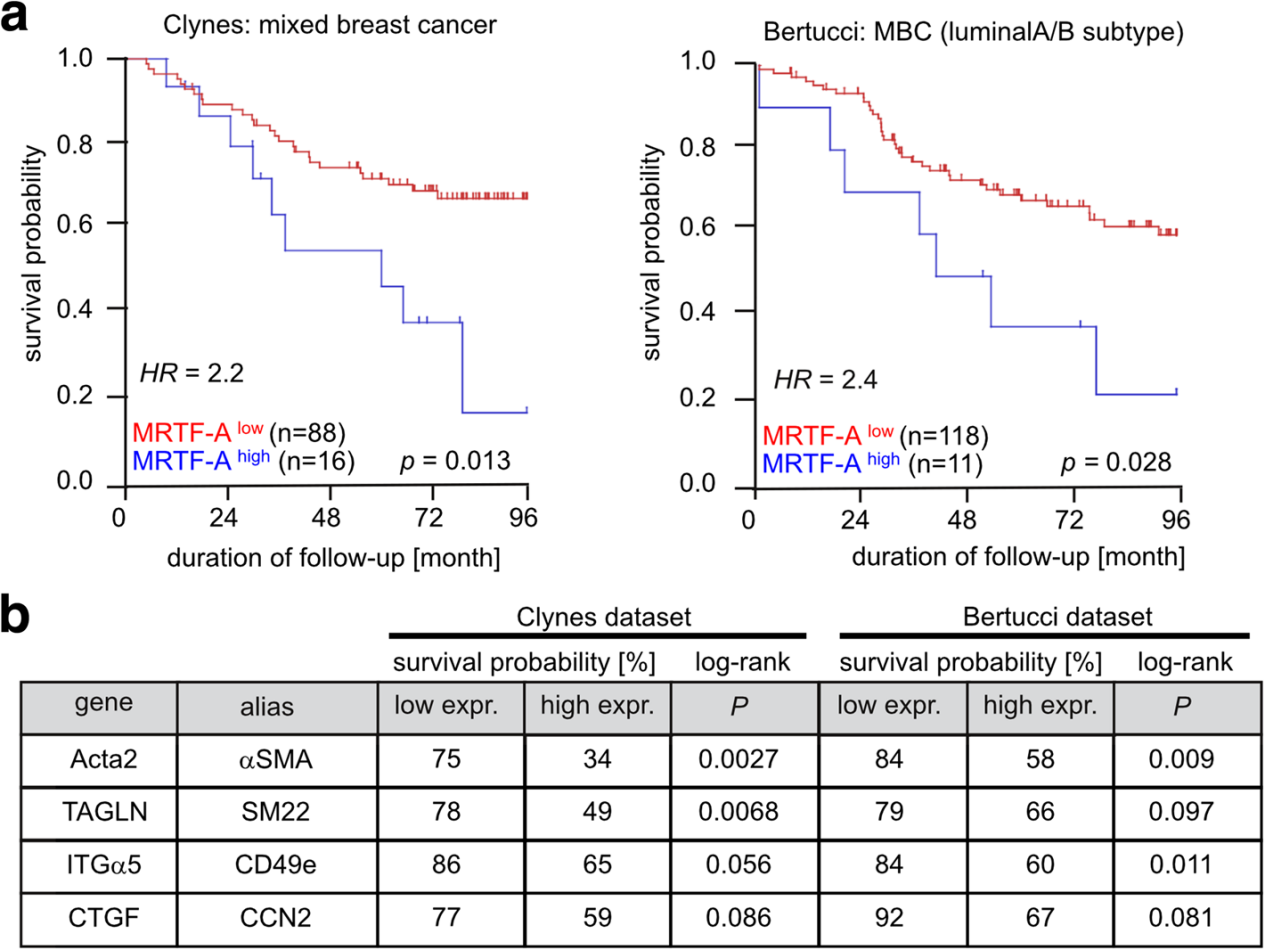
Survival rates in breast cancer patients with high expression of myocardin-related transcription factor (*MRTF*)-A and its target genes. Microarray mRNA expression datasets of Clynes [24] and Bertucci [25] were analyzed using the R2 microarray and visualization platform software. **a** Kaplan–Meier plots for 96 months of follow up of 104 patients with mixed types of breast cancer (Clynes) or a luminal A/B subset of patients with medullary breast cancer (n = 129; Bertucci), using the *p* value scanning method. Relative hazard ratios *(HR)* for high MRTF-A expression (*blue lines*) were calculated following age adjustment and Cox regression analysis. *Dashes* indicate censored patients. **b** Five-year survival probability in patients with low or high expression of the MRTF target genes *ACTA2*, *TAGLN*, *ITGA5* and *CTGF*. Data were extracted from Kaplan–Meier graphs of the datasets indicated: *p* values were calculated for significant differences, using the log-rank test

Finally, we investigated whether MRTF-A is relevant in human breast cancer. Using the R2 visualization platform, two different breast cancer datasets were analyzed for association between MRTF-A or MRTF-A target gene expression and patient survival. In cohorts from the Clynes [24] and Bertucci [25] datasets, high MRTF-A mRNA abundance was significantly associated with lower 8-year survival among patients (Fig. 9a). The age-adjusted HR was 2.42 (95% CI 1.13–5.15) in 104 patients with mixed types of breast cancer [24]. In the Bertucci dataset of medullary breast cancer of the luminal A and B subtype, the age-adjusted HR was 2.32 (95% CI 1.01 − 5.34) [25]. Thus, patients with high MRTF-A expression had more than twofold increase in the risk of cancer mortality in comparison to those with low MRTF-A expression (Fig. 9a).

Since MRTF-A expression does not necessarily represent its transcriptional activity, the known MRTF-A target genes *ACTA2*, *TAGLN*, *ITGA5* and *CTGF* were also analyzed in these datasets. The 5-year survival rate was generally lower among the patients with high expression of *ACTA2*, *TAGLN*, *ITGA5* and *CTGF* (Fig. 9b). Whilst these initial analyses indicated a considerable trend amongst patients, the differences were not always significant according to gene-specific log-rank tests, possibly due to small patient numbers. To this end, however, the data suggest that high MRTF-A activity tend to result in poor prognosis for breast cancer patients.

## Discussion

The human mammary epithelial cell line MCF10A is a non-transformed, widely used model to analyze differentiation and transformation when cultured in extracellular matrix components. The formation of acini within 14 days of 3D culture enables the analysis of proliferation, apoptosis, polarization, cell-cell and cell-matrix interactions [17]. Thus, several studies have analyzed signaling of oncogenes and tumor suppressors in the acinar structures [18, 27]. Using this model, we showed that tightly controlled activity of MRTFs is required for normal acinar morphogenesis. While MRTF knockdown dramatically reduces acini size and prevents lumen formation, overexpression of MRTFs causes luminal filling during morphogenesis, elevated spheroid size and upregulation of EMT markers. However, the MCF10A model is limited to the analysis of luminal cells and cannot characterize MRTF effects on myoepithelial differentiation.

### MRTFs ensure acinar morphogenesis

We observed nuclear accumulation of MRTF-A, transient MRTF/SRF reporter activation and induction of MRTF target gene expression at day 5 of acinar morphogenesis, suggesting that early stages of acini formation stimulate the actin-MRTF signaling pathway (Fig. 1c, d, e). Several lines of evidence link the actin cytoskeleton to epithelial acinar morphogenesis and potentially involve MRTF regulation. SiRNA-dependent knockdown of the F-actin organizers Rac1 and Cdc42 or expression of their dominant negative variants inverts polarity in kidney epithelial cysts [28, 29]. Inhibition of myosin II, a major downstream effector of Rho-kinase, causes a loss of peripheral F-actin bundles and outgrowth of microtubules [30]. Interestingly, cytochalasin-D-mediated destruction of F-actin in day-3 acini inhibits the random and rotational cell movement required for 3D morphogenesis [31].

In turn, modulation of MRTF activity leads to rearrangment of F-actin in acinar epithelial cells, which normally is localized predominantly at the lateral contact zone and apical sides (Figs. 2 and 3). Based on their various cytoskeletal target genes, MRTF-A and MRTF-B depletion is known to reduce actin expression and migration [32]. Actin dynamic is thus both a cause and a consequence of altered MRTF/SRF activity. Therefore, we propose that MRTF-A activity is simultaneously induced by and required for the actin-controlled formation of polarized epithelial acini. However, the early proliferative defects upon MRTF depletion and the late morphological alterations in MRTF-A-overexpressing acini suggest distinct activities of MRTF at different time points, highlighting the importance of precise temporal control during morphogenesis.

### MRTF-dependent control of proliferation via cell cycle regulators

Depletion of MRTF-A leads to very small acini without lumen and is accompanied by considerable upregulation of the CKI p21/Waf1, p27/Kip1 and hypophosphorylation of Rb. A rescue experiment showed that the size reduction, lumen formation and organization of the cortical actin are restored by MRTF-A re-expression, whereas MRTF-B cannot fully compensate for the loss of MRTF-A (Figs. 3 and 4). Vice versa, downregulation of p21/Waf1, p27/Kip1 and Rb hyperphosphorylation by MRTF overexpression provoke luminal filling (Figs. 2 and 6a, b). In line with this, we previously observed a modest anti-proliferative effect in MRTF-depleted NIH3T3 fibroblasts evoked by deregulated cell cycle control, including increased p21/Waf1 expression [33]. Deletion of MRTF-A/B also prevents xenograft growth, associated with increased cdkn2a (p16) expression and hypophosphorylation of Rb [34]. Therefore, we propose that MRTFs control cell cycle progression by regulating CKI and the phosphorylation of Rb in a context-dependent manner. Indeed, proliferation and apoptosis still occur simultaneously at day 14 in MRTF-overexpressing spheroids, whilst proliferation and apoptosis ceases in control acini after day 10. Since BIM-associated apoptosis of the inner cells in MCF10A acini caused by elevated Myc expression can be attenuated by dominant active MRTF-A [35, 36], we conclude that luminal filling upon MRTF overexpression is caused by MRTF-dependent deregulation of myc-associated apoptosis.

### MRTF-A and MRTF-B overexpression overcomes anoikis

Loss of integrin binding to ECM ligands induces anoikis, which causes lumen formation during acinar morphogenesis of MCF10A [18, 37]. However, MRTF overexpression results in anchorage-independent growth, and cell death is neither detectable in early morphogenesis nor restricted to the inner cell mass at later times, suggesting deregulated anoikis (Figs. 7 and 8). Alteration of integrin expression is one strategy for avoiding anoikis. Disruption of integrin α6/β4 activation influences polarity [38, 39]. In spheroids overexpressing MRTF-A or MRTF-B, we noted a loss of integrin α6 localization, and lamininV was no longer restricted to the basal lamina (Figs. 2 and 8b). Instead, the fibronectin receptor integrin α5, known as a MRTF-A target gene [7], was upregulated and localized predominantly at the basal side (Fig. 8). The regulation of integrin-dependent adhesion and the dynamics of the actin cytoskeleton is bidirectional: assembly and maturation of focal adhesions is regulated by cytoskeletal forces, whilst F-actin polymerization and bundling is modulated by the growing focal adhesions [40]. In our morphogenesis experiments in MRTF-overexpressing spheroids no additional external force was applied. Thus, we speculate that MRTFs control anoikis by directing the expression and/or localization of integrins. Alternatively, integrin signaling towards MRTFs might be altered, or EMT is induced, generating anoikis-resistant cells in MRTF-overexpressing spheroids.

### Loss of mechanosensing

We observed stiffness-dependent regulation of luminal clearance. At early time points, acinar morphogenesis was accelerated by addition of collagen (Fig. 7a). Adding collagen from days 5.5 to 8 pronounced MRTF/SRF reporter activation and its shutdown afterwards (Fig. 7c). This suggests that luminal clearance is controlled by MRTF activity, which may act as both a regulator and a sensor. Healthy cells continuously sense force by integrins and adapt their behavior to the microenvironment. Force generation is also involved in mammary gland differentiation [41]. It has been shown that RhoA, the upstream regulator of MRTF-A/B, plays a significant role in branching morphogenesis, which is regulated via traction or the clefting force of growing buds [17]. In contrast, the permanent rigidity of the ECM disrupts MCF10A acini morphology [42]. The suspected mechanotransduction sensors are MRTF and TAZ/YAP of the Hippo pathway [8, 43]. Recently, functional interaction and crosstalk between MRTF and TAZ has been suggested [6, 8, 44]. Thus, both MRTF and TAZ might cooperate in sensing ECM rigidity by collagen treatment. The promotion of luminal clearance in untransformed MCF10A acini is not observed in MRTF-overexpressing spheroids. Regulation of the stiffness by reducing/increasing the matrigel concentration also did not affect the phenotype of MRTF-overexpressing spheroids or MRTF-A knockdown acini (data not shown). Therefore, the MRTF-dependent integrin switch or the cortical actin organization may overcome external mechanosensing.

### MRTF induce EMT

Overexpression of MRTF-A or MRTF-B regulates EMT marker proteins as vimentin, Snai2 and epithelial marker protein E-cadherin in MCF10A cells. These results raise the possibility that elevated MRTF activity leads to a cellular dedifferentiation process, which contributes to the loss of mammary epithelial architecture. Recently, an oncogenic function of MRTF-A/B in normal pancreatic cells was reported, caused by increased stem cell formation and promotion of EMT [45]. MRTF-A has also frequently been implicated in transforming growth factor (TGF)β1-induced EMT and myofibroblast transition [19, 46]. The regulation of EMT marker proteins, together with the observed luminal filling, anchorage-independent growth and deregulated cell cycle/apoptosis indicates a transforming potential of MRTF-A or MRTF-B in MCF10A cells. Indeed, the luminal filling is an event in early-stage carcinoma [18]. Combined regulation of apoptosis and proliferation is also well-known for many oncogenes. Therefore, we speculate that MRTFs promote tumor initiation in breast epithelia. Moreover, increasing tension due to tumor growth and compressive force is characteristic of breast malignancies [17]. Loss of mechanoreciprocity promotes progression of disease [41]. In this context, we presented experimental hints that mechanotransduction induced by external stiffness is overruled by aberrant MRTF activity. Whether MRTF-A indeed plays an important role in human breast cancer pathogenesis awaits future clinical investigations. To this end, our limited epidemiological analysis of preexisting breast cancer databases suggests that high expression of MRTF-A and its target genes increases the hazard ratio and decreases 5-year survival rates, respectively.

## Conclusions

Precise temporal control of MRTFs is required for normal morphogenesis of MCF10A mammary acini. MRTF-A knockdown represses proliferation during morphogenesis. These effects are rescued by re-expression of MRTF-A, and partially by MRTF-B. Conversely, overexpression of MRTF-A and MRTF-B impaired apico-basal polarity, promoted cell growth and survival and deregulated expression of epithelial and mesenchymal markers. Analysis of breast cancer databases showed that high expression of MRTF-A and known target genes was associated with decreased patient survival. Together with the increasing experimental evidence suggesting a tumor-promoting function, MRTFs may therefore serve as promising targets for future approaches in cancer therapy.

## Additional files

Additional file 1: Figure S1. MRTF-A expression and activity. **a** MRTF-A and MRTF-B expression during acinar morphogenesis of MCF10A cells for 15 days. Protein extracts were prepared from recovered acini and blotted with antibodies as indicated. As control, GAPDH protein is shown. **b** MRTF-A and MRTF-B protein expression was examined in MCF10A and NIH3T3 cells. All western blots are representative of two independent experiments. (TIFF 8243 kb)

Additional file 2: Figure S2. Characterization of MCF10A cells overexpressing MRTFs. **a** MRTF-A and MRTF-B protein expression in stably transduced pools of cells overexpressing MRTF-A or MRTF-B or vector-infected (mock) and parental control cells. A representative immunoblot with specific antibodies against MRTF-A, MRTF-B and GAPDH as a control is shown. Quantified relative protein amounts of MRTF-A were normalized to parental MCF10A cells. **b** Stably transduced cells were transiently transfected under serum-starved conditions, treated after 24 h with horse serum for 7 h if indicated and analyzed for MRTF/SRF reporter activity. *Error bars* SEM (n = 3): *p < 0.05, **p < 0.01, ***p < 0.001 (Student’s *t* test). (TIFF 14057 kb)

Additional file 3: Figure S3. MRTF-A knockdown impair acini formation. **a** Immunfluorescence showing MRTF-A, MRTF-B, lamininV, F-actin of shControl and shMRTF-A#3 cells. Representative mid plane sections are shown and nuclei are stained in *blue. Scale bar* 20 μm. **b** Quantification of acini size of bright field images. Data shown are quantification of at least 100 acini (day 14) per cell line from each of three independent experiments. The *box* represents the interquartile range and the *middle line* represents the median; *whiskers* extend to 5th and 95th percentiles. ShControl and shMRTF-A#3 cells were examined for MRTF-A and MRTF-B protein expression (**c**) and SRF-dependent promoter activity (**d**). **e** Immunfluorescence showing Ki67 staining of shControl and shMRTF-A#3 cells. Representative mid plane sections are shown and nuclei are stained in *blue. Scale bar* 20 μm. *Error bars* SEM (n = 3): *p < 0.05 (Student’s *t* test). (TIFF 20363 kb)

Additional file 4: Figure S4. MRTF-A re-expression in MRTF-A knockdown cells rescue proliferation during morphogenesis. Immunfluorescence showing K67 staining of shControl + GFP, sh#1 + MRTF-A and sh#2 + MRTF-A cells. Representative midline sections are shown and nuclei are stained in *blue. Scale bar* 20 μm. (TIFF 5624 kb)

Additional file 5: Figure S5. Impact of MRTF overexpression on acinar morphogenesis of MCF10A cells. Quantification of cleaved caspase-3-positive acini at day 14; 30 acini were quantified from each of three different experiments of the MRTF-overexpressing and control MCF10A cells. *Error bars* SEM (n = 3): *p < 0.05, ***p < 0.001 (Student’s *t* test). (TIFF 7078 kb)

Additional file 6: Figure S6. Zeb1 (**a**) and Twist (**b**) expression in MRTF-overexpressing MCF10A cells. Quantitative RT-PCR. *Error bars* SEM (n = 3): *p < 0.05 (Student’s *t* test). (TIFF 8243 kb)

## Abbreviations

CKI: Cyclin-dependent kinase inhibitors
CTGF: Connective tissue growth factor
ECM: Extracellular matrix
EGF: Epidermal growth factor
EMT: Epithelial-mesenchymal transition
FCS: Fetal calf serum
GAPDH: glyceraldehyde-3-phosphate dehydrogenase
GFR: Growth factor-reduced
HR: Hazard ratio
miRNA: MicroRNA
MKL: Megakaryoblastic leukemia
MRTF: Myocardin-related transcription factor
Rb: Retinoblastoma protein
shCrtl: Small hairpin RNA control
shRNA: Small hairpin RNA
SNAI1: Snail family transcriptional repressor 1
SRF: Serum response factor
TAGLN: Transgelin
Zeb1: Zinc finger e-box binding Homeobox 1
