# Supplementary figures and images for: Tightly controlled MRTF-A activity regulates epithelial differentiation during formation of mammary acini
Source: Breast Cancer Res. 2017 Jun 7;19:68. doi: 10.1186/s13058-017-0860-3 (PMC5463372; doi:10.1186/s13058-017-0860-3)

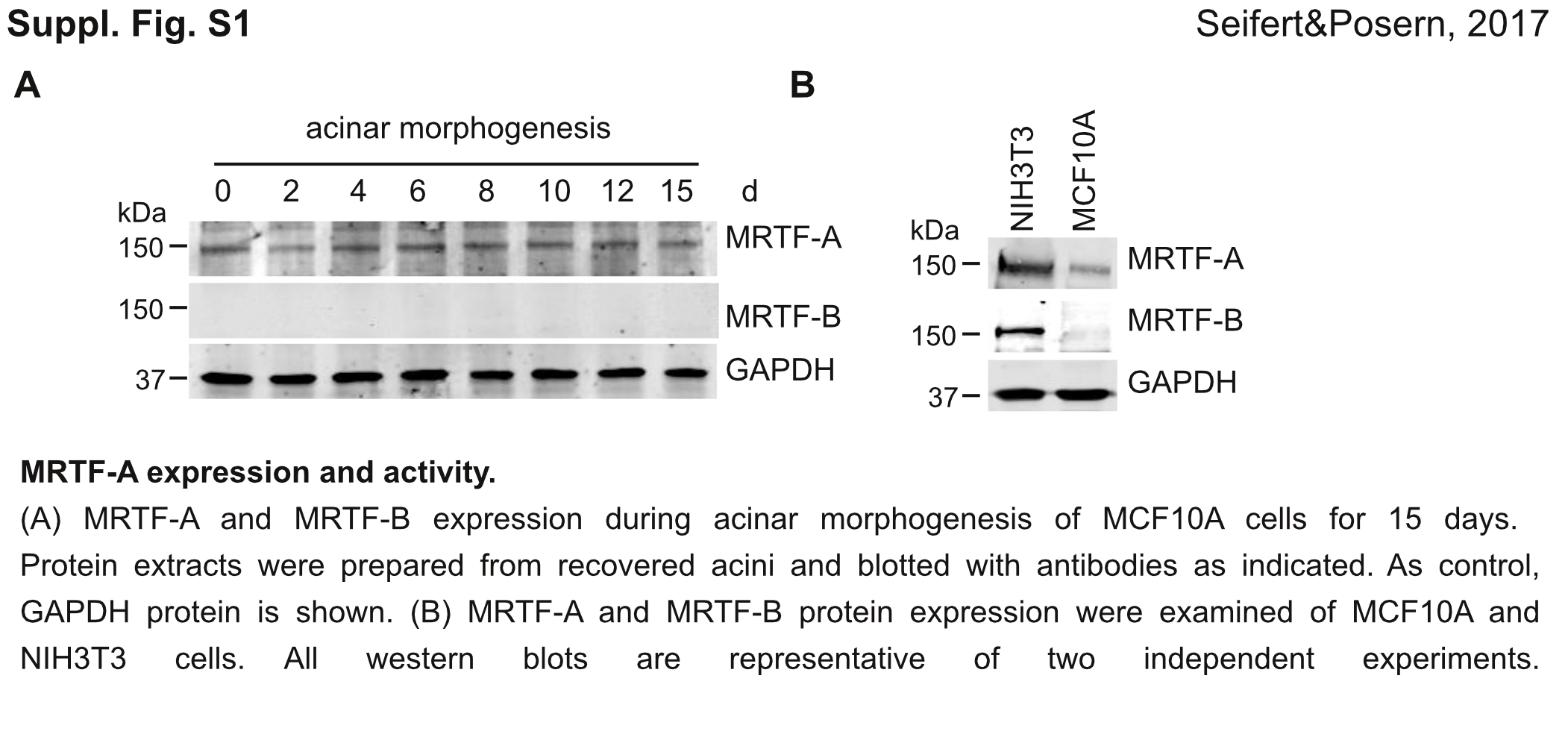

Supplement: Supplementary file 1 — MRTF-A expression and activity. a MRTF-A and MRTF-B expression during acinar morphogenesis of MCF10A cells for 15 days. Protein extracts were prepared from recovered acini and blotted with antibodies as indicated. As control, GAPDH protein is shown. b MRTF-A and MRTF-B protein expression was examined in MCF10A and NIH3T3 cells. All western blots are representative of two independent experiments. (TIFF 8243 kb) [file 13058_2017_860_MOESM1_ESM.tiff]

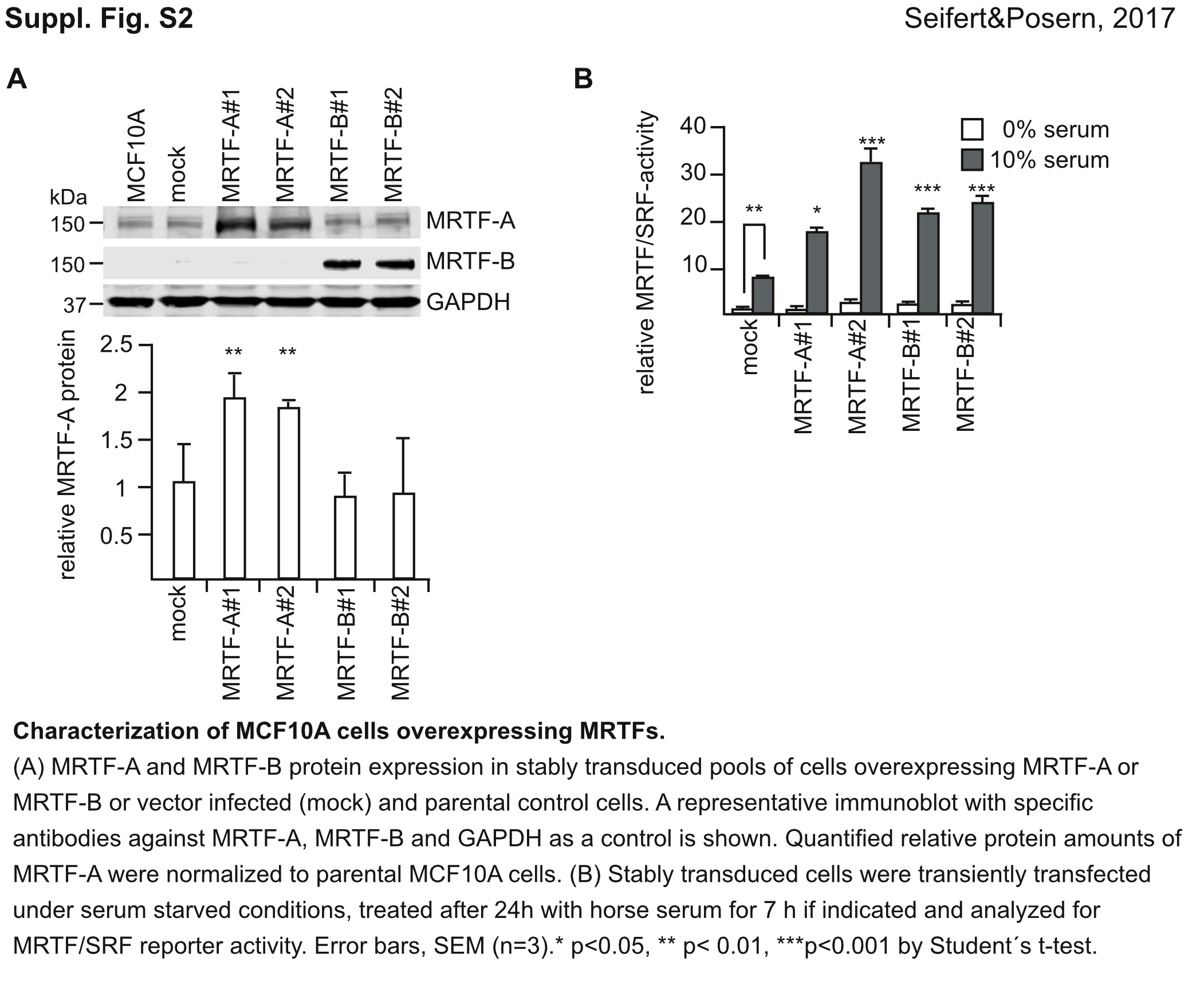

Supplement: Supplementary file 2 — Characterization of MCF10A cells overexpressing MRTFs. a MRTF-A and MRTF-B protein expression in stably transduced pools of cells overexpressing MRTF-A or MRTF-B or vector-infected (mock) and parental control cells. A representative immunoblot with specific antibodies against MRTF-A, MRTF-B and GAPDH as a control is shown. Quantified relative protein amounts of MRTF-A were normalized to parental MCF10A cells. b Stably transduced cells were transiently transfected under serum-starved conditions, treated after 24 h with horse serum for 7 h if indicated and analyzed for MRTF/SRF reporter activity. Error bars SEM (n = 3): *p < 0.05, **p < 0.01, ***p < 0.001 (Student’s t test). (TIFF 14057 kb) [file 13058_2017_860_MOESM2_ESM.tiff]

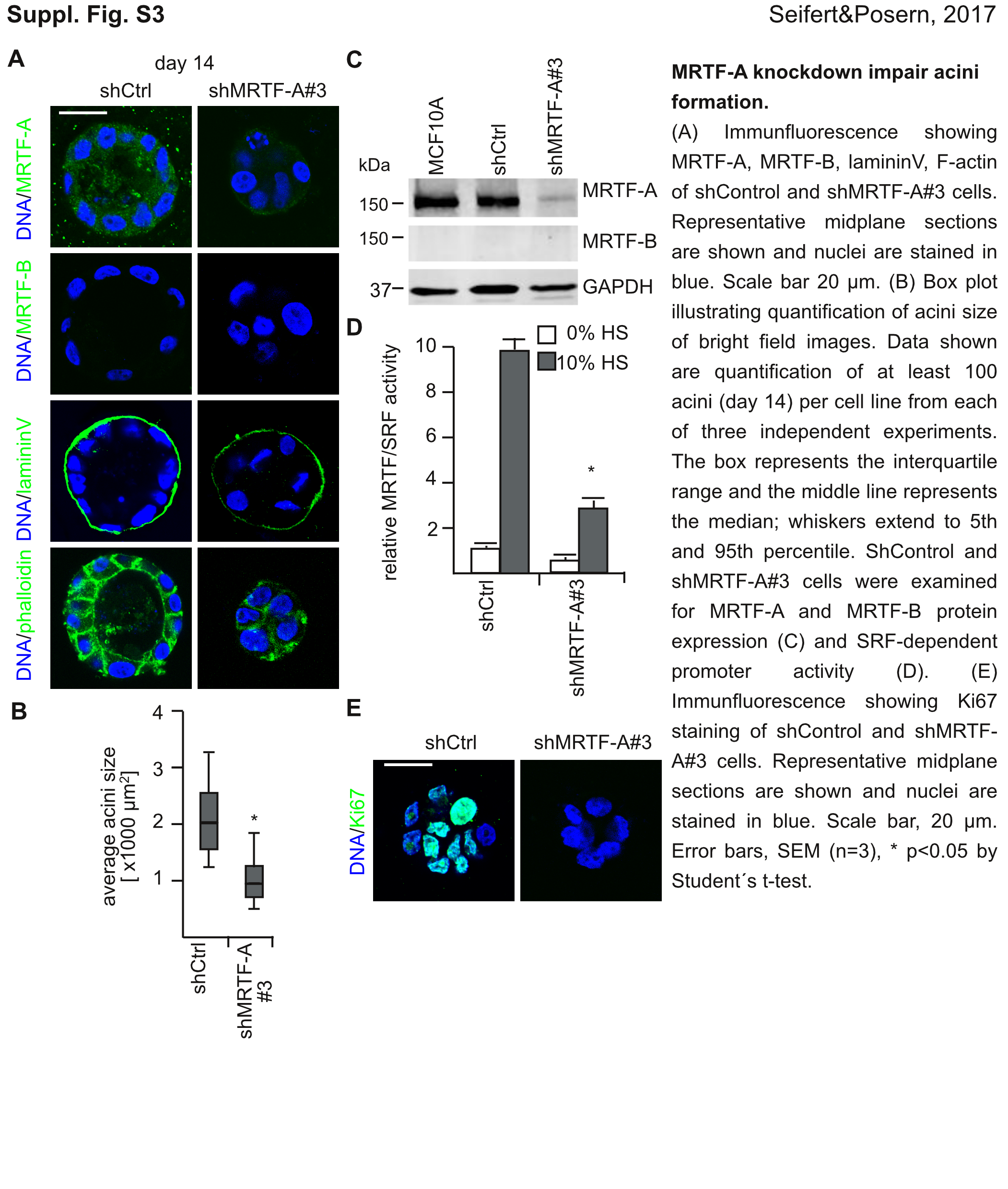

Supplement: Supplementary file 3 — MRTF-A knockdown impair acini formation. a Immunfluorescence showing MRTF-A, MRTF-B, lamininV, F-actin of shControl and shMRTF-A#3 cells. Representative mid plane sections are shown and nuclei are stained in blue. Scale bar 20 μm. b Quantification of acini size of bright field images. Data shown are quantification of at least 100 acini (day 14) per cell line from each of three independent experiments. The box represents the interquartile range and the middle line represents the median; whiskers extend to 5th and 95th percentiles. ShControl and shMRTF-A#3 cells were examined for MRTF-A and MRTF-B protein expression (c) and SRF-dependent promoter activity (d). e Immunfluorescence showing Ki67 staining of shControl and shMRTF-A#3 cells. Representative mid plane sections are shown and nuclei are stained in blue. Scale bar 20 μm. Error bars SEM (n = 3): *p < 0.05 (Student’s t test). (TIFF 20363 kb) [file 13058_2017_860_MOESM3_ESM.tiff]

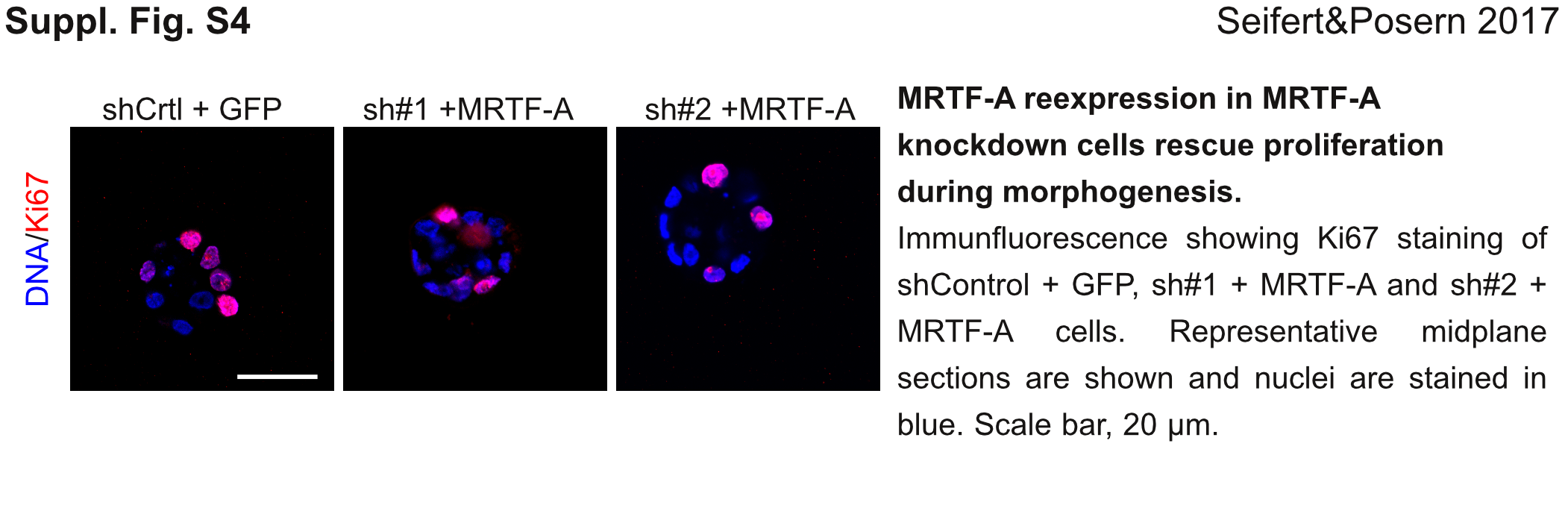

Supplement: Supplementary file 4 — MRTF-A re-expression in MRTF-A knockdown cells rescue proliferation during morphogenesis. Immunfluorescence showing K67 staining of shControl + GFP, sh#1 + MRTF-A and sh#2 + MRTF-A cells. Representative midline sections are shown and nuclei are stained in blue. Scale bar 20 μm. (TIFF 5624 kb) [file 13058_2017_860_MOESM4_ESM.tiff]

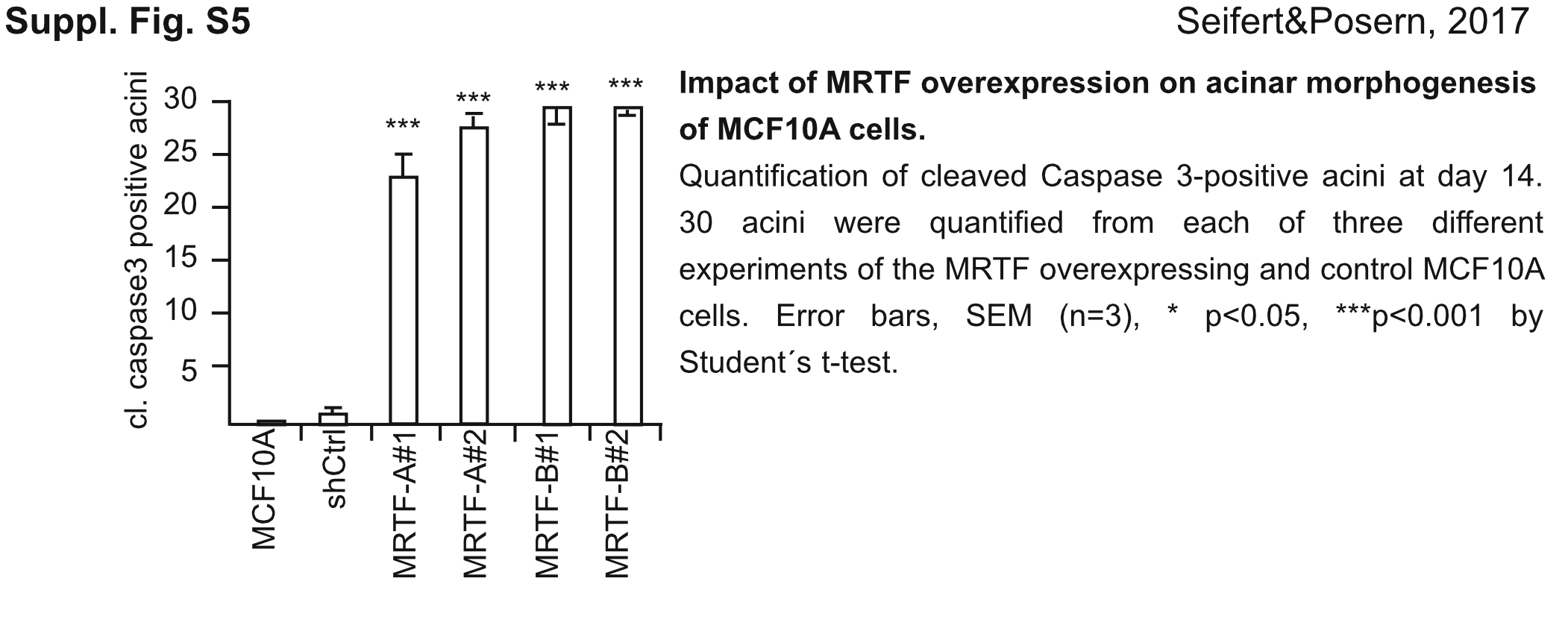

Supplement: Supplementary file 5 — Impact of MRTF overexpression on acinar morphogenesis of MCF10A cells. Quantification of cleaved caspase-3-positive acini at day 14; 30 acini were quantified from each of three different experiments of the MRTF-overexpressing and control MCF10A cells. Error bars SEM (n = 3): *p < 0.05, ***p < 0.001 (Student’s t test). (TIFF 7078 kb) [file 13058_2017_860_MOESM5_ESM.tiff]

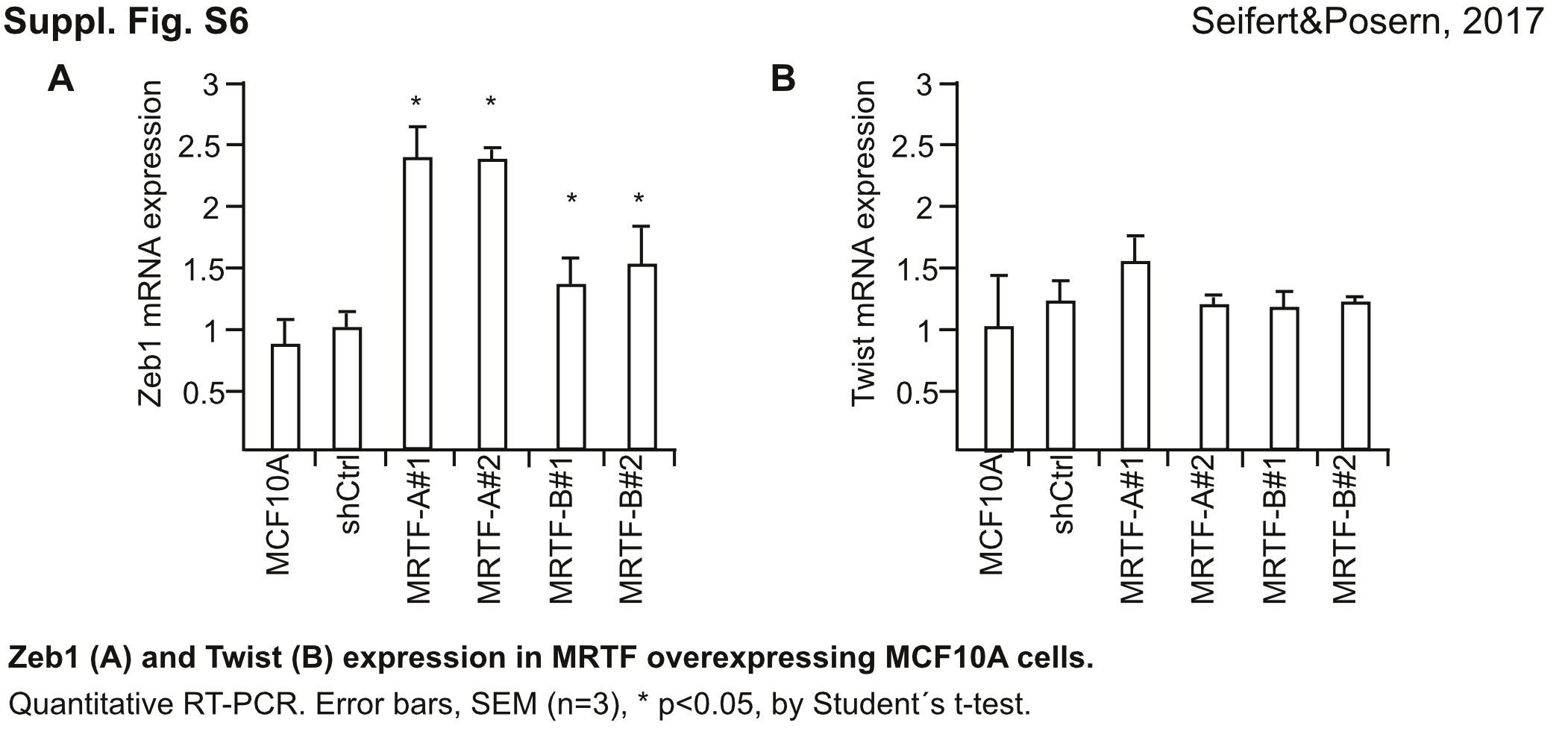

Supplement: Supplementary file 6 — Zeb1 (a) and Twist (b) expression in MRTF-overexpressing MCF10A cells. Quantitative RT-PCR. Error bars SEM (n = 3): *p < 0.05 (Student’s t test). (TIFF 8243 kb) [file 13058_2017_860_MOESM6_ESM.tiff]
